# Supplementary material for: Emergent exchange-driven giant magnetoelastic coupling in a correlated itinerant ferromagnet
Source: Nat Phys. 2025 Jun 17;21(8):1243–9. doi: 10.1038/s41567-025-02893-x (PMC12343299; doi:10.1038/s41567-025-02893-x)
Supplement: Supplementary file 1 — Supplementary Figs. 1–19 and Sections 1–11. [file 41567_2025_2893_MOESM1_ESM.pdf]

nature physics

Supplementary information https://doi.org/10.1038/s41567-025-02893-x

Emergent exchange-driven giant magnetoelastic coupling in a correlated itinerant ferromagnet

In the format provided by the
authors and unedited

CONTENTS

S1. Point spectra in zero magnetic field 2

S2. Magnetostriction from DFT 3

S3. Electronic structure for parallel and antiparallel spin configuration 4

A. Models without spin-orbit coupling 5

B. Model with spin-orbit coupling 7

S4. Temperature dependence of bulk coercive field 10

S5. Magnetic field dependence - high fields 11

S6. Magnetic ground state configuration 12

S7. Temperature dependence of switching field 13

S8. Large scale topographies and terrace size 16

S9. Domain wall pinning in 2D 18

A. Area swept out by the domain wall 19

B. Thermal activation 20

S10. Magnetostriction 22

A. Magnetostriction loops 22

B. Detection of magnetostriction jumps in magnetic field ramps 22

C. Detection of magnetostriction jumps in time traces 25

D. Absence of bias-dependence in jumps 28

References 30

S1. POINT SPECTRA IN ZERO MAGNETIC FIELD

Upon cooling down the sample to 80 mK in zero magnetic field, two sharp peaks, P_{I} and P_{II} are observed (black curve in Fig. S1). After ramping the field to above 1 T and back to 0 T, the peak positions of both P_{I} and P_{II} are the same as in the zero-field cooled case. The same is observed when ramping the field to below -1 T and back to 0 T. It confirms that the magnetizations of the surface layer and bulk are parallel upon zero-field cooling, and establishes the shape of the tunneling spectra for a parallel configuration of the magnetization of the surface layer and bulk.

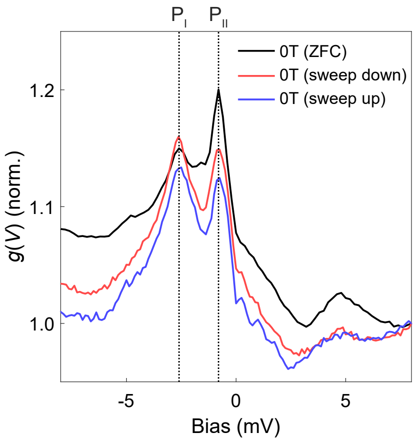

FIG. S1. **Tunneling spectra for parallel alignment of surface and bulk magnetizations.** Tunneling spectra $g(V)$ after cooling the sample without an applied magnetic field (black), and at 0 T after applying $B_z > 1$ T (red) and $B_z < -1$ T (blue). The energies of peaks P_{I} and P_{II} are the same for all three cases, consistent with the alignment of the magnetization of the surface layer and bulk in the same direction upon cooling down with no magnetic field applied. All spectra were normalized to 1 at 8 mV and were taken at a temperature of $T = 80$ mK. The black curve is an average spectrum of a grid of 16×16 spectra over a clean area of 1.7×1.7 nm^2 ($V_{\mathrm{set}} = 10$ mV, $I_{\mathrm{set}} = 500$ pA, $V_{\mathrm{L}} = 200$ μV). The red curve was taken in the same area as the blue curve and they are both the result of the average of an 8×8 grid over an 1×1 nm^2 area ($I_{\mathrm{set}} = 450$ pA).

S2. MAGNETOSTRICTION FROM DFT

In real materials, the change in energy between antiferromagnetic and ferromagnetic ground states as a function of atomic distance is often more complex than the behaviour suggested by the Bethe-Slater curve, for example, due to the orbital character of the wave functions involved.[1] Therefore, we have performed density functional theory calculations of slabs and model bilayer systems to determine the exchange interaction and its influence on the electronic structure.

The large unit cell of $Sr_4Ru_3O_{10}$ makes DFT calculations and structural relaxation for the full triple-layered structure computational demanding. One can show, however, that to a very good approximation, the electronic structure and key properties can be captured using just a single layer of Sr_2RuO_4, including octahedral rotations, instead of the full triple layer[2]. The electronic structure of the triple layer consists of three replica of that of the single layer[3]. We therefore discuss here results for calculations for both, the full crystal structure of $Sr_4Ru_3O_{10}$

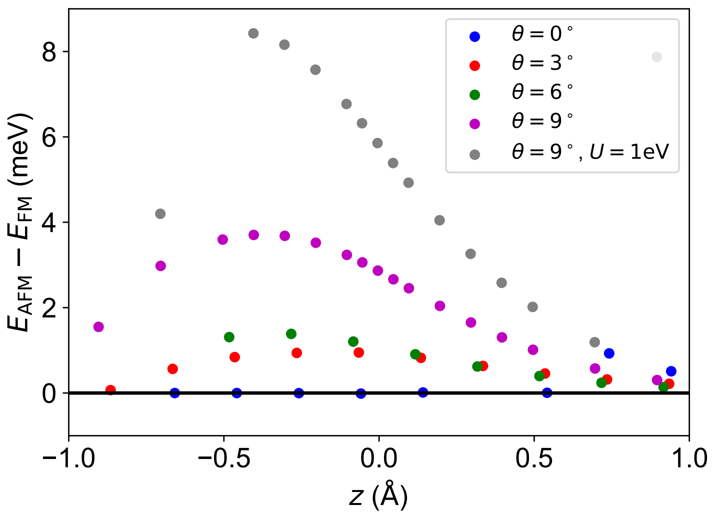

FIG. S2. **Exchange interaction for two Sr_2RuO_4 layers.** The exchange interaction $E_{AFM} - E_{FM}$ between two Sr_2RuO_4 layers with different octahedral rotations θ in a slab with vacuum above and below is shown as a function of interlayer separation z. $z = 0$Å corresponds to the energy minimum for the case of ferromagnetic interaction. The exchange interaction is shown for $\theta = 0°$, $3°$, $6°$, and $9°$. For $9°$, we show in addition the exchange interaction for the calculation including a Hubbard-U term, with $U = 1$eV.

as well as of two layers of Sr_2RuO_4.

In Fig. S2, we show a model calculation of the exchange interaction for different distances between two layers of Sr_2RuO_4 and for different octahedral rotations in these layers. For these calculations, we have not performed a structural relaxation, but taken the crystal structure of Sr_2RuO_4 and introduced the octahedral rotation and interlayer separation. We find that at equilibrium distance, the interaction is ferromagnetic. The exchange force is expected to result in an outward relaxation of the surface layer for antiferromagnetic coupling. Both, increased octahedral rotation θ as well as increased Hubbard U results in a larger exchange interaction. The qualitative behaviour is consistent with that expected from the Bethe-Slater curve in the ferromagnetic tail.

We have performed extensive studies of the relaxation for ferromagnetic and antiferromagnetic alignment of adjacent layers of both Sr_2RuO_4 and $Sr_4Ru_3O_{10}$ to establish the influence of the magnetic configuration on the structure. Because of the tiny energies involved, we have compared the results from different DFT codes and using different exchange correlation functionals. The studies have been performed using Quantum Espresso and VASP, and using PBE and PBEsol as exchange correlation functionals. The key results from these studies are: (a) we find an energy difference between ferromagnetic and antiferromagnetic configurations of adjacent layers of Sr_2RuO_4 or $Sr_4Ru_3O_{10}$ of a few meV, consistently favouring the ferromagnetic configuration (compare fig. S2). (b) when relaxing the surface layer of slabs of either Sr_2RuO_4 or $Sr_4Ru_3O_{10}$ (while fixing the in-plane lattice constant, as is the case in the experiment), we find consistently that the surface layer relaxes outward by about 50fm in the antiferromagnetic configuration compared to the ferromagnetic configuration. (c) inclusion of U in LDA+U schemes tends to increase the energy difference between antiferromagnetic and ferromagnetic spin configurations, stabilizing the ferromagnetic configuration.

S3. ELECTRONIC STRUCTURE FOR PARALLEL AND ANTIPARALLEL SPIN CONFIGURATION

In order to understand the origin of the shift in the VHs observed in tunneling spectra, as shown in fig. 2 in the main text, we have studied the electronic structure of the model systems

of two layers of Sr_2RuO_4 introduced in section S2.

A. Models without spin-orbit coupling

To explain the changes in the tunneling spectra found between parallel and antiparallel spin configurations, we show in fig. S3 model calculations for a minimal model based on two single layers of Sr_2RuO_4 with an octahedral rotation of $\theta = 9^\circ$. The tight-binding model for the two layers is obtained from a non-magnetic DFT calculation performed using VASP and with the PBE functional to describe the exchange-correlation energy. We use $8 \times 8 \times 1$ **k**-points and an energy cut-off of 600eV. The electronic structure for the two free-standing layers is projected onto a tight-binding model using Wannier90[4]. We then introduce the same modifications as described in ref. [2], except that here the spin-orbit coupling is initially set to zero for clarity. The magnetism is introduced at the tight-binding level. In Fig. S3(a), we show the resulting band structure for ferromagnetic alignment with a band crossing between spin-majority and spin-minority bands highlighted by the red circle. This band crossing is lifted by spin-orbit coupling and results in a VHs similar to the one previously identified by STM and ARPES.[2] For antiferromagnetic arrangement, this band crossing is lifted (fig. S3(b)), because the bands acquire the same spin character in adjacent layers. Once spin-orbit coupling is included, the hybridization seen here adds to the hybridisation due to spin-orbit coupling. We note that in these model calculations, the crossing is slightly above the Fermi energy, because the models are based on a different octahedral rotation compared to ref. 2.

In fig. S4, we show that the same arguments hold true for a full DFT calculation using Quantum Espresso for two layers of $Sr_4Ru_3O_{10}$. Fig. S4(a) shows the band structure for the ferromagnetic case, calculated without spin-orbit coupling. The red circle highlights the part of the electronic structure which is seen in tunneling spectra and which is highlighted in fig. S3(a). Without spin-orbit coupling, these bands just cross because of their opposite spin character. For antiferromagnetic arrangement between the adjacent layers (fig. S4(b)), a small hybridization gap forms. While the precise contribution of the different bands and hybridizations is difficult to disentangle, this mechanism provides an explanation for the characteristic changes we observe.

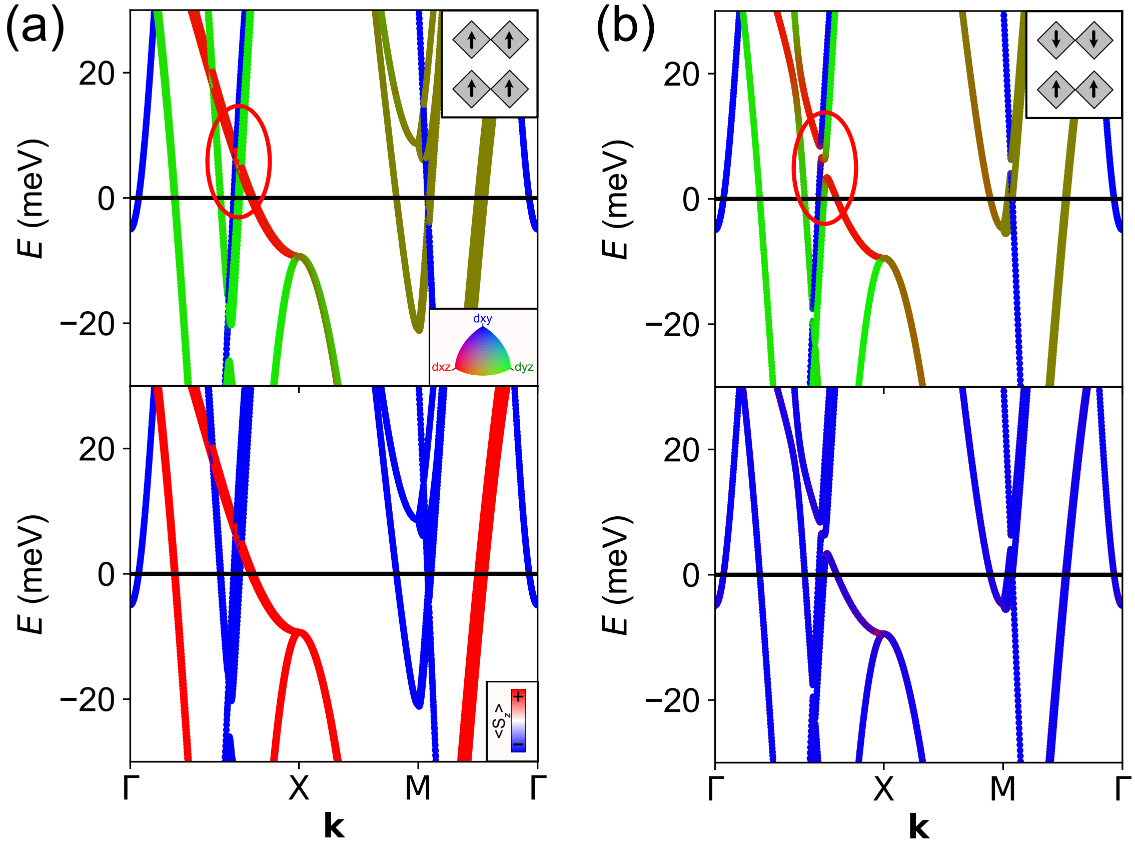

FIG. S3. **Electronic structure for parallel and antiparallel spin configuration for a minimal model.** Band structure obtained from a paramagnetic tight-binding model for the band structure of two layers of Sr_2RuO_4 with magnetism added manually. The bandstructure is shown for (a) ferromagnetic (FM) and (b) antiferromagnetic (AFM) alignment of the adjacent layers. For FM alignment, there is no hybridization between the spin-majority band of $d_{xz/yz}$ character and the spin-minority bands of d_{xy} and $d_{xz/yz}$. (b) Due to the finite interlayer coupling, in the antiferromagnetic case, a small hybridization gap opens at the crossing between the bands of spin-minority and majority character, because of the opposing spin configuration in the adjacent layers. Note though that spin-orbit coupling would open a hybridization gap even in the ferromagnetic case, so the hybridization seen here in the antiferromagnetic case adds to the hybridization gap due to spin-orbit coupling. Upper panels show orbital character (blue: d_{xy}, red/green d_{xz}, d_{yz}), lower panels spin character of the bands.

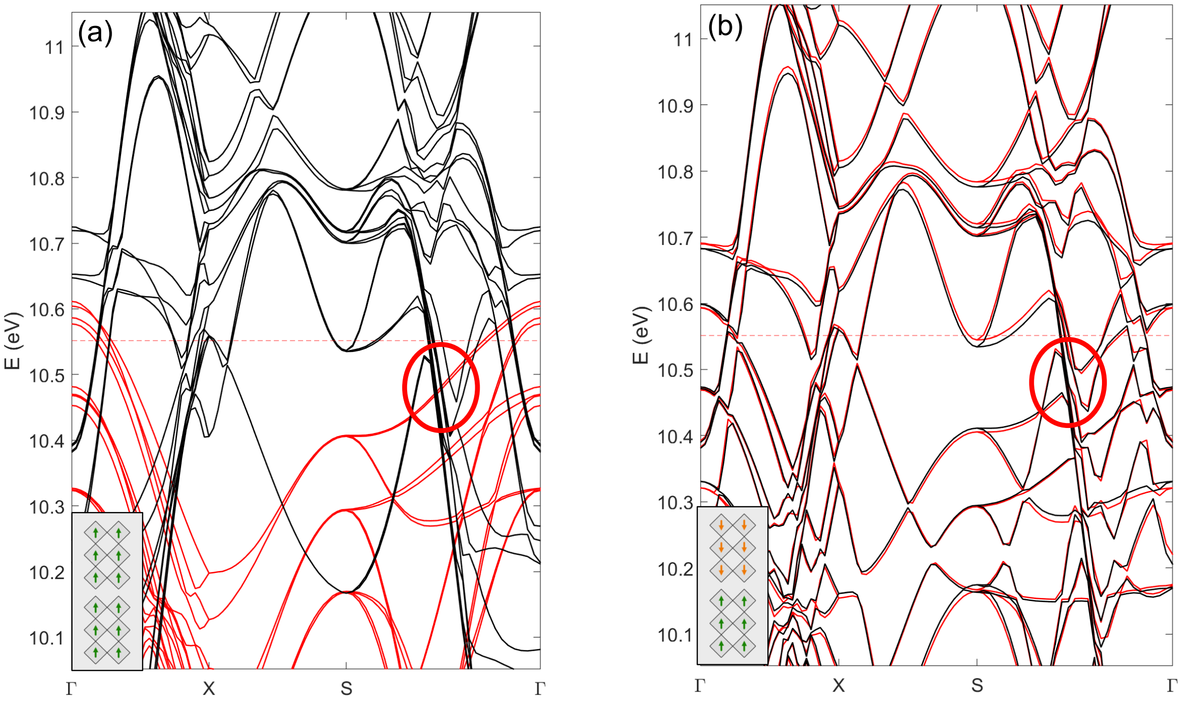

FIG. S4. Bandstructure of $Sr_4Ru_3O_{10}$ for (a) ferromagnetic and (b) antiferromagnetic configuration of two adjacent triple layers. The red circle highlights the hybridization gap that is seen in tunneling spectra. The calculation here is done without spin-orbit coupling. Black and red indicate spin minority and majority bands, respectively.

B. Model with spin-orbit coupling

As we have shown previously, spin-orbit coupling in combination with the octahedral rotation has important consequences for the band structure of both, the surface layer of Sr_2RuO_4 and for bulk $Sr_4Ru_3O_{10}$.[2, 5] Here, we introduce spin-orbit coupling into the simplified tight-binding model with two layers of Sr_2RuO_4 introduced in the previous section and shown in fig. S3. To facilitate comparison with experiment, we have shifted the Fermi energy such that the upper Van Hove singularity aligns with the experimentally observed position (Fig. 2b of the main text). Initially we consider the system with spin-orbit coupling and spins parallel to the c-axis, noting, however, that there is experimental evidence that the spins are slightly tilted away from the c

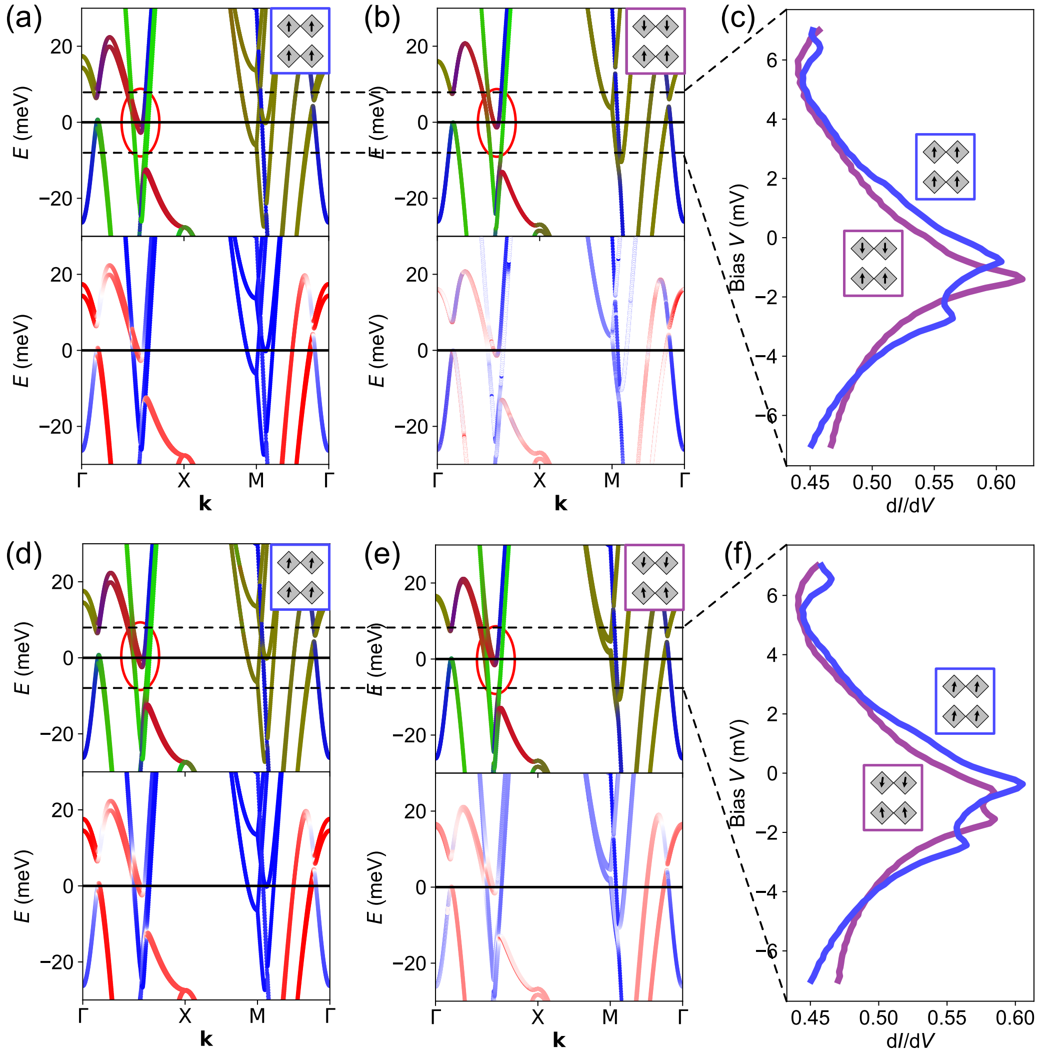

FIG. S5. **Minimal model for magnetization direction induced changes in spectra.** Band structures of the minimal model with spin-orbit coupling consisting of two layers of Sr_2RuO_4 with an octahedral rotation of $\theta = 9°$ and with the chemical potential adjusted so that the energy of the VHs is consistent with experiment, for (a) ferromagnetic and (b) antiferromagnetic alignment and spins along the c-axis. The red circle highlights the spin-orbit coupling induced Van Hove singularity. (c) simulated tunneling spectra for the ferromagnetic (blue) and antiferromagnetic (purple) alignment. Panels (d-f) show the same graphs as (a-c), however now with the spins tilted by 8° degree away from the c axis. The simulated spectra in f now reproduce the experimental data in both cases, i.e. for ferromagnetic and antiferromagnetic configurations. The colorbars in (a, b, d, e) are the same as in Fig. S3(a).

axis.[6–8] In fig. S5a-c, we show the band structure (a) for the ferromagnetic ground state with spins aligned parallel to the c-axis, (b) with antiferromagnetic alignment and in (c) simulated tunneling spectra in the bias voltage range of ± 7mV shown for the experiments. For spins aligned along the c-axis, one can see a single Van-Hove singularity for antiferromagnetic alignment, and two Van-Hove singularities for ferromagnetic alignment. The latter is a direct consequence of the interlayer coupling of the Van Hove singularity itself, resulting in a bilayer splitting. In contrast with the experimental data, only one peak is seen in the case of antiferromagnetic alignment. While there may be a number of reasons for why the minimal model presented here fails to account for the pair of peaks in the antiferromagnetic case, one possible reason (backed up by experimental evidence[6–8]) would be if the spins are not aligned perfectly with the c axis, but slightly canted away from the c axis in the same direction for both layers. Panels d-e show a calculation where the spins have been tilted by 8° degree away from the c axis. While this does not result in any noticeable change for the ferromagnetic case, it results in a small hybridization between the Van Hove singularities in the two layers in the antiferromagnetic case, producing a small splitting as is seen in the experimental data (compare, e.g., Fig. 2b of the main text).

S4. TEMPERATURE DEPENDENCE OF BULK COERCIVE FIELD

We have measured the bulk magnetization of single crystals of $Sr_4Ru_3O_{10}$ from the same batch as the crystals used for the STM measurements using the Vibrating Sample Magnetometry option of a Physical Property Measurement System by Quantum Design. We obtain the hysteresis loops by sweeping the magnetic field between ±1 T, and extract the coercive field H_c. Figure S6(a) shows the hysteresis loops measured at decreasing temperatures, from 20 K to 2 K. In Fig. S6(b), we plot the coercive field determined from the loops in fig. S6(a). In the main text in fig. 3g, we show the average coercive field at each temperature as open circles.

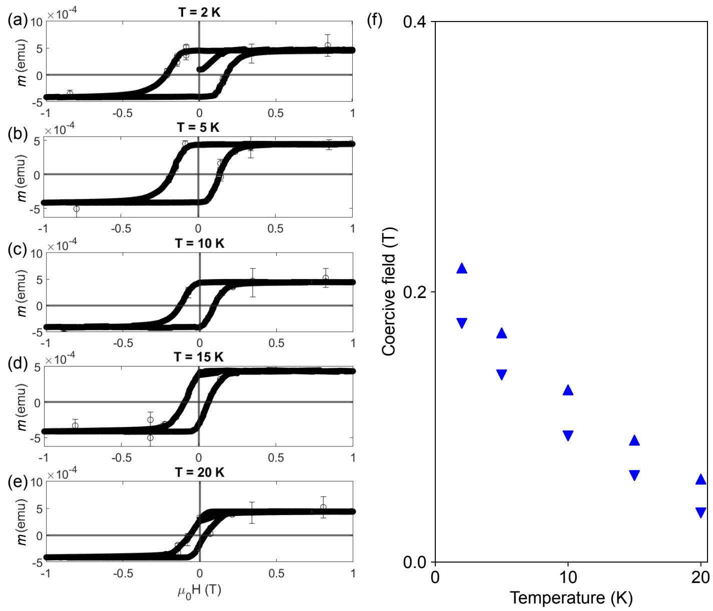

FIG. S6. **Coercive field from magnetization measurements.** (a-e) Magnetization m vs field $\mu_0 H$ loops of $Sr_4Ru_3O_{10}$ measured at temperatures between T = 2K and T = 20K between B_L = +1T and −1T. (f) Coercive field extracted from the M-H loops in (a)-(e) plotted as a function of temperature. A slight asymmetry between positive and negative fields is observed which is likely a consequence of the sample not being fully polarized at ±1T. Error bars in (a)-(e) are standard errors obtained from the regression to extract magnetizations.

S5. MAGNETIC FIELD DEPENDENCE - HIGH FIELDS

To determine the spin character of the peaks identified in the tunneling spectra, we have measured $g(V)$ as a function of magnetic field B_z from 0 T to 13.5 T, applied parallel to the crystallographic c-axis of the sample. Figure S7 shows a series of tunneling spectra taken from 0 T to 13.5 T in steps of 1 T. Both peaks P_I and P_{II} move towards lower energy, as expected for a VHs of majority-spin character. We note that the slope remains constant within the errors of our measurements throughout the field range (Figure S7b), suggesting that the magnetization of the sample remains always aligned with the c-axis.

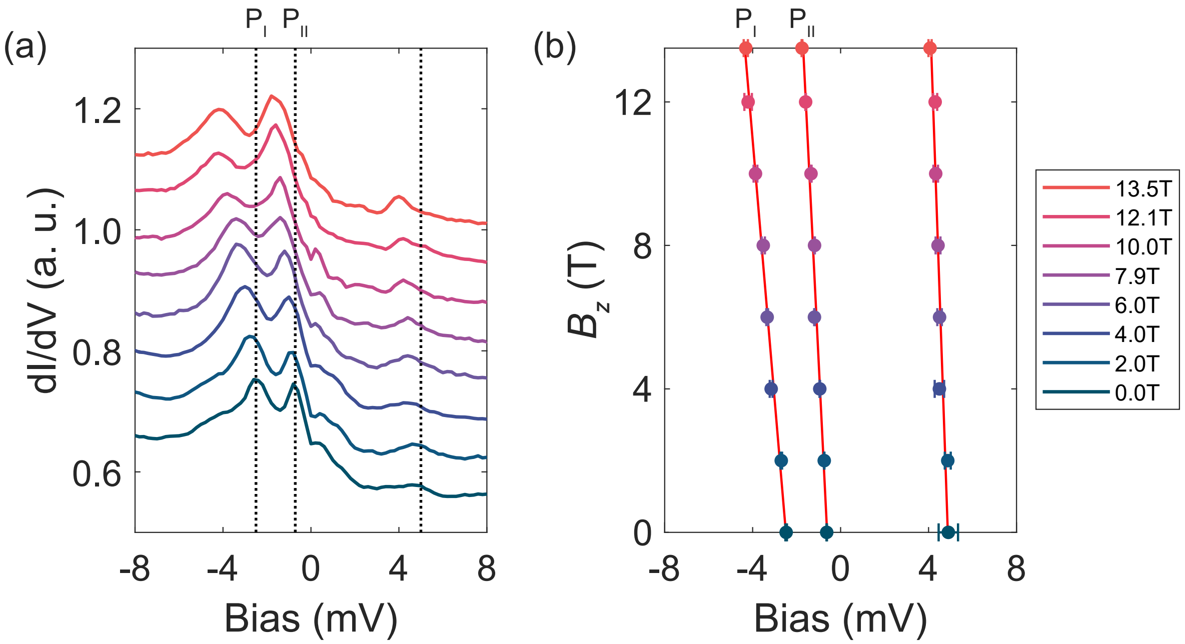

FIG. S7. **Magnetic field dependence.** (a) High resolution tunneling spectra $g(V)$ as a function of magnetic field. The spectra are offset vertically for clarity ($V_{\text{set}} = 10$ mV, $I_{\text{set}} = 500$ pA, $V_{\text{L}} = 200$ μV). (b) Peak positions extracted from a fit of three Lorentzian peaks to the spectra in (a), error bars are the 95% confidence intervals. P_I and P_{II} shift towards lower energies as expected for a majority spin character. Red lines show a linear fit to the data.

S6. MAGNETIC GROUND STATE CONFIGURATION

To demonstrate that it is indeed the entire top triple layer that flips its magnetization, rather than just the top layer of the surface triple layer, we have performed DFT calculations for ferromagnetic and antiferromagnetic alignment of the magnetizations in neighbouring triplet layers, as well as for only one of the three layers flipping its magnetization. The ferromagnetic configuration is the ground state, and is 4.5meV lower than the antiferromagnetic state of two triplet layers with opposite magnetization of the two triplet layers in the unit cell, showing that the two are indeed very close in energy. Consistent with the small interlayer coupling, the band structure (Fig. S8(a, b)) changes only very subtly. For the case of one of the three layers within a triplet having a switched magnetization (Fig. S8(c), we get a significantly higher energy of about 200meV, rendering this state highly unlikely to be realized. Furthermore, the band structure shows significant changes, on the scale of the hopping term of the Ru atoms in the top surface layer and in the first sub-surface layer. Such large changes are not consistent with the small changes seen in tunneling spectra also in a wider energy range (compare Figure S19).

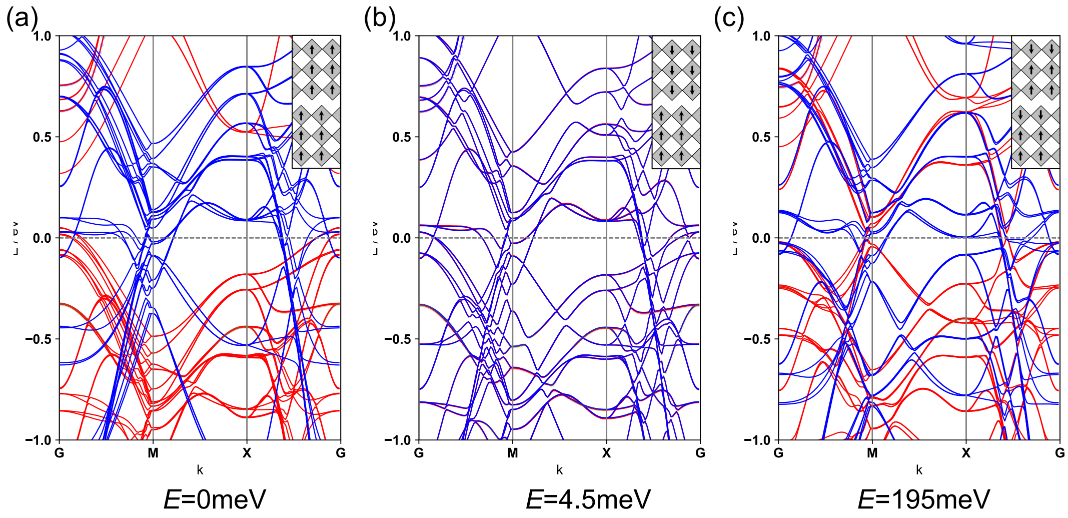

FIG. S8. **Band structure and energies of magnetic configurations.** (Band structure for (a) ferromagnetic configuration, (b) antiferromagnetic configuration of neighbouring triple layers and (c) the magnetization of one of the three layers in a triple layer flipped. Between (a) and (b) there are only subtle differences in the band structure, whereas (c) shows a significantly different band structure. E is the energy relative to the ferromagnetic configuration (a). While the antiferromagnetic configuration is only 4.5meV higher in energy, configuration (c), where only one of the layers in a triple layer is flipped, has an energy almost 200meV higher, rendering this configuration highly unlikely.

S7. TEMPERATURE DEPENDENCE OF SWITCHING FIELD

Figure S9(a-f) show the tunneling spectra for the two configurations of the surface and bulk magnetizations for all temperatures measured in this study. Cycling the magnetic field from 1.3 T to −1.2 T and back to 1.3 T for all temperatures allowed us to extract the evolution of peak P_I for temperatures $T = 80$ mK to $T = 2.9$ K by fitting two Lorentzian peaks to the spectra. At higher temperatures, $T = 6$ K to $T = 14$ K, the two peaks cannot be distinguished, however the characteristic change can still be seen in the spectra and detected in the energy of only a single peak fitted to the data. The evolution shown in panels (g-l) of Figure S9 was used to extract the switching fields of the bulk (lower field) and surface layer (high field), shown in Fig. 3(g) of the main manuscript.

From the fitting of Lorentzian functions with two peaks at temperatures $T = 80$ mK to $T = 2.9$ K, we can also plot the evolution of peak P_{II}, identified previously as corresponding to a saddle point vHs[2]. Figure S9 (m-o) shows the peak position of P_{II} as a function of magnetic field, for the same cycles as before. At temperature $T > 1$K, the field dependence of P_{II} is difficult to assess, whereas for $T = 80$ mK, it is consistent with the surface layer retaining its magnetization direction with a slight shift in the peak energy, which is however close to the size of the error bars.

Figure S10(a-f) show colour plots of all the $g(V)$ curves taken while cycling the magnetic field back and forth from 1.3 T to -1.2 T, from which the points of Figure S9(g-l) were extracted. The curves shown in Figures S9 and S10 are the result of averaging the $g(V)$ spectra over a grid taken on a clean surface. Figure S11 shows topographies taken before the first point and the last point of the cycle measured at each temeprature. The red squares in the panels of Figure S11 indicate the area over which the $g(V)$ grids were taken and over which the $g(V)$ curves were averaged over to obtain the curves shown in Figures S9 and S10. These grids were taken over a few unit cells to obtain an average $g(V)$ curve that reflects the $g(V)$ within a unit cell.

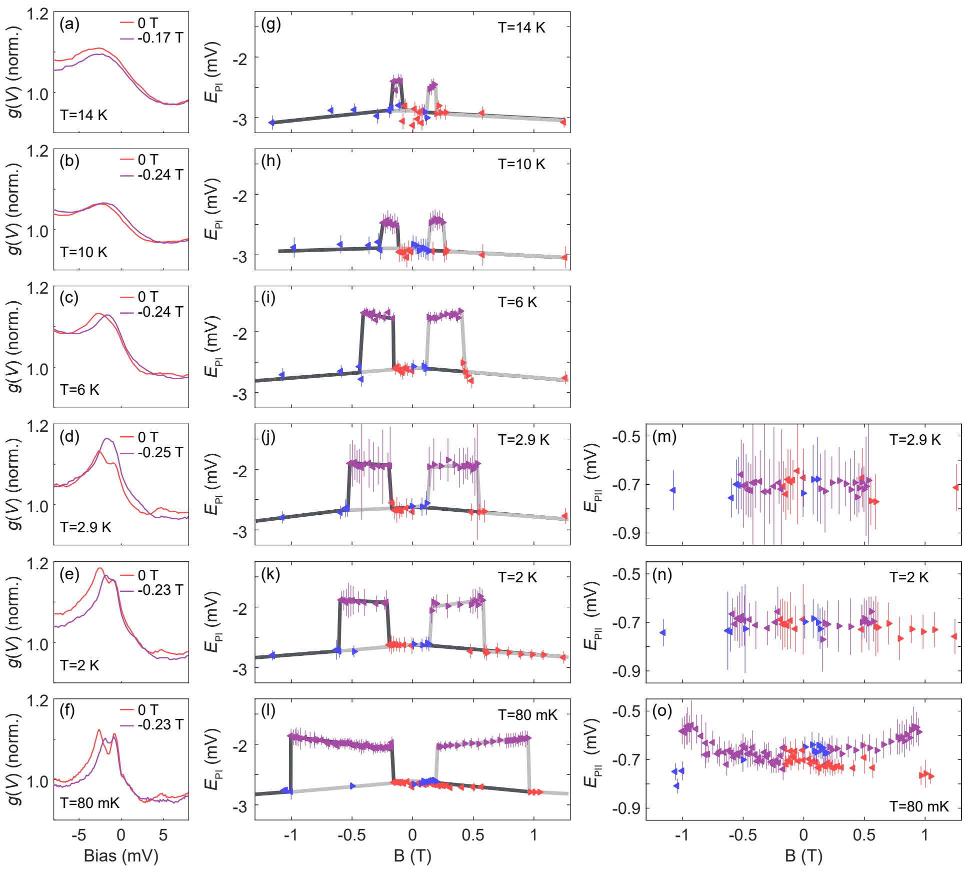

FIG. S9. **Additional data for temperature dependence of switching.** (a-f) Differential conductance spectra $g(V)$ at different temperatures: 14 K (a), 10 K (b), 6 K (c), 2.9 K (d), 2 K (e), and 80 mK (f). The red curves show spectra for a parallel configuration of surface and bulk magnetizations at 0 T and the purple curves show the spectra for an anti-parallel configuration at -0.23 T. (g-l) Position of $\mathrm{P_I}$, E_{PI}, as a function of magnetic field B_z by cycling the field from 1.3 T to -1.2 T and back to 1.3 T at 14 K (g), 10 K (h), 6 K (i), 2.9 K (j), 2 K (k) and 80 mK (l). Each point was obtained by fitting Lorentzian functions with two peaks (j-l) or with one peak (g-i). (m-o) Peak position of $\mathrm{P_{II}}$, E_{PII}, as a function of field, after cycling the magnetic field from 1.3 T to -1.2 T and back to 1 T, at 2.9 K (m), 2 K (n) and 80 mK (o). These were taken from the same fits as $\mathrm{P_I}$ in (j, k, l). Each curve at each field is the result of averaging spectra recorded on an 16×16 grid over a (1.5×1.5) nm^2 area for $T = 80$ mK and on an 8×8 grid over a (1×1) nm^2 area for the other temperatures ($V_{\mathrm{set}} = 10$ mV, $I_{\mathrm{set}} = 450$ pA. $V_{\mathrm{L}} = 400$ μV at 14 K, 10 K and 6 K; $V_{\mathrm{L}} = 250$ μV at 2.9 K and 2 K; $V_{\mathrm{L}} = 125$ μV at 80 mK). The error bars in (g-o) are the 95% confidence intervals from the fitting.

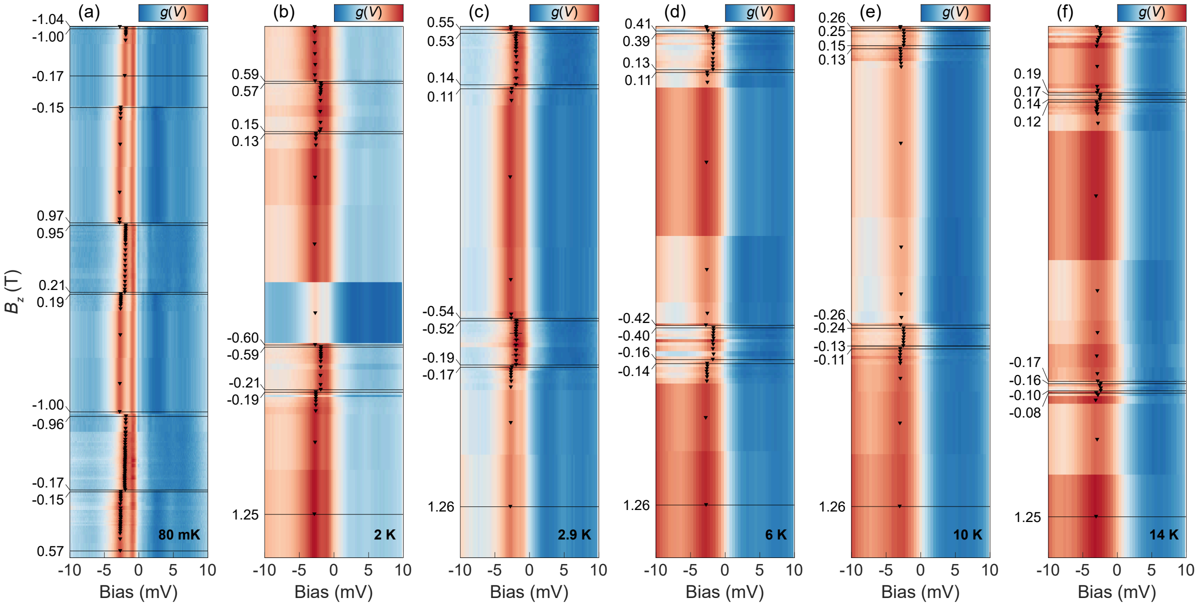

FIG. S10. **Complete $g(V, T, B)$ data set for all temperatures measured.** (a-g) Tunneling spectra $g(V, T, B)$ taken as the field B was cycled back and forth between 1.3 T to −1.2 T, at all temperatures T measured in this study. The points shown in Fig. S9(g-l) were extracted from these curves and are shown as black downward triangles on top of each colour plot for the corresponding temperature. The error bars are the 95% confidence intervals.

S8. LARGE SCALE TOPOGRAPHIES AND TERRACE SIZE

The cleaving of $Sr_4Ru_3O_{10}$ single crystals produces SrO-terminated surfaces as previously reported[2, 9], and consistent with the cleaving of single crystals of other members of the Ruddlesden-Popper series of the strontium ruthenates[10, 11]. Terraces have often sizes of multiple hundred nanometers. A topographic image recorded in an area of $(1.7\mu\text{m})^2$ is shown in Fig. S12. It shows a few line defects, but no terraces. From tunneling spectra taken on either side of the line defects, there is no evidence that they pin domain walls similarly strongly as step edges do, i.e. we always see the surface layer with the same magnetization direction relative to the bulk either side of the line defects, suggesting that they do not act as strong pinning sites.

Some cleaves show evidence for the presence of a low concentration ($< 1\%$) of CO molecules

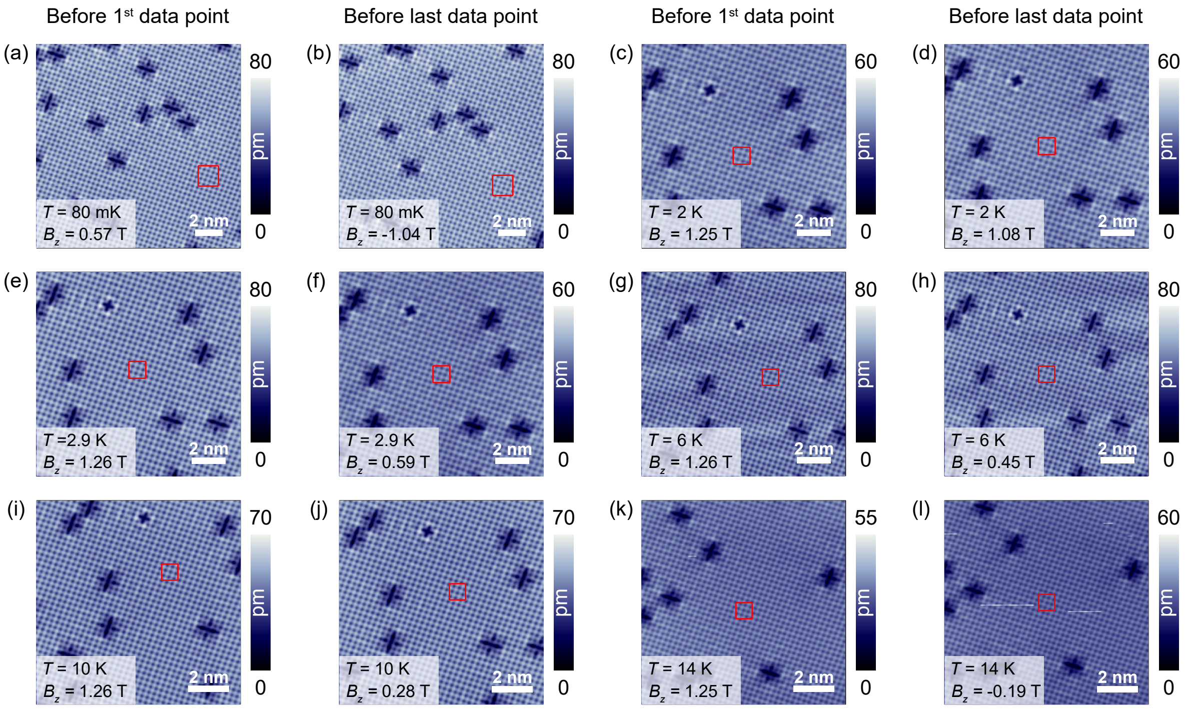

FIG. S11. **Series of topographies taken at all temperatures measured.** Topographies taken before the first (a,c,e,g,i and k) and last (b,d,f,h,j and l) data points shown in Fig. S10, for all temperatures measured: 80 mK (a,b), 2 K (c,d), 2.9 K (e,f), 6 K (g,h), 10 K (i,j) and 14 K (k,l). The red squares in each panel indicate the area over which each $g(V)$ spectra shown in Fig. S10 was averaged over. For all topographies a polynomial background of second order was subtracted, and the topographies taken at 6 K in addition have the average of each line removed (setpoint conditions for all topographies: $V_{\mathrm{set}} = 10$ mV, $I_{\mathrm{set}} = 450$ pA).

on the surface. These are seen as dark crosses in Fig. S11.

S9. DOMAIN WALL PINNING IN 2D

This note provides a derivation of the temperature dependence of the coercive field for a 2D ferromagnet assuming strong pinning of a domain wall, following the theory by P. Gaunt [12] and extending the recent derivation presented in [13]. We consider the example of a 180° domain wall pinned by three equally spaced pinning sites (see fig. S13). Application of a small magnetic field causes the domain wall to bow in order to maximize the magnetization whilst remaining pinned by the pinning sites. At larger fields, the domain wall is expected to unpin at the center[12]. The pinning of domain walls modifies the coercive field required to reverse the

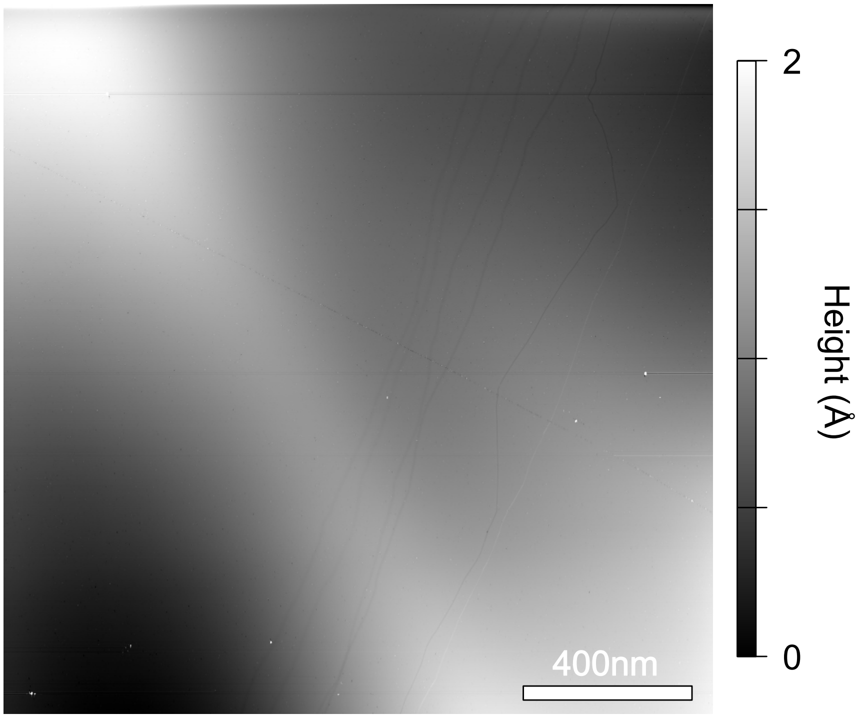

FIG. S12. **Large-scale topographic imaging.** Topographic image recorded in zero magnetic field over an area of $1.68 \times 1.68 \mu m^2$ with 2048×2048 pixels over more than 28h, showing a large area without any terraces. To remove vertical piezo drift, a polynomial background has been subtracted, as well as removing the average of each line. Step edges would show up with a height of $\sim$ 15Å, an order of magnitude larger than the scale bar shown. ($T = 1.8$K, $V = -7.5$mV, $I = 50$pA).

magnetization of the sample magnetization. Even if the applied field does not reach the coercive field, thermal activation can allow for unpinning of the domain wall, thus lowering the effective coercive field. In this note, we calculate this temperature dependence.

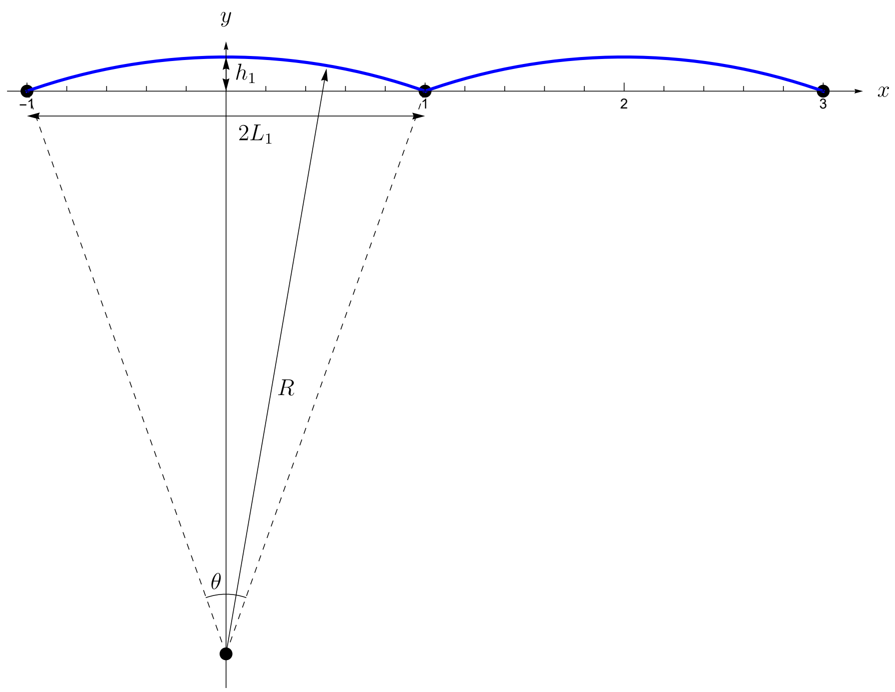

FIG. S13. Domain wall pinning geometry considered in this note.

A. Area swept out by the domain wall

We begin by calculating the area swept out by the unpinned domain wall. Consider three pinning sites, equally spaced, with the distance between sites, $d = 2L_1$ (fig. S13). The area of a circular segment with subtending angle θ and of radius of curvature R is given by $A = R^2(\theta - \sin\theta)/2$. The subtending angle is $\theta = 2\sin^{-1}(L_1/R)$. The total area, prior to breakaway is thus

$$A_{\text{prior}} = 2A = \left[2\sin^{-1}\left(\frac{L_1}{R}\right) - \sin\left(2\sin^{-1}\left(\frac{L_1}{R}\right)\right)\right] R^2. \tag{S1}$$

The area after the breakaway from the central pin can then be found. The radius and angle remain the same as before, but the secant length is doubled

$$A_{\text{post}} = \left[2\sin^{-1}\left(\frac{2L_1}{R}\right) - \sin\left(2\sin^{-1}\left(\frac{2L_1}{R}\right)\right)\right]\frac{R^2}{2}. \tag{S2}$$

We now proceed to calculate the change in area following the unpinning. To deal with the trigonometric functions, we expand in the small quantity L_1/R, assuming that $R \gg L_1$. The change in area is given by

$$\Delta A \approx 4R^2\left(\frac{L_1}{R}\right)^3 = 4\frac{L_1^3}{R} \tag{S3}$$

to third order in L_1/R. It is straightforward to show that also in the two-dimensional case the relation $HM = \gamma/R$, originally derived for the 3D case[14], holds, where M is moment per unit area and γ is the domain wall energy per unit length. From this we find that the change in area induced by the field, H, is

$$\Delta A = 4HM\frac{L_1^3}{\gamma}. \tag{S4}$$

On depinning from one site, one can expect the domain wall to propagate further until it reaches a new pinning site. If it re-attaches at most to one new pinning site while sweeping the area ΔA, it will be able to continue propagation, resulting in a steady state criterion

$$\rho\Delta A \leq 1. \tag{S5}$$

where ρ is the number of pinning sites per unit area. We will proceed using $\rho\Delta A = 1$, which provides an upper limit for the coercive field. Rearranging for the inter-pin distance, $2L_1$ we have

$$2L_1 = 2\left(\frac{\gamma}{4\rho HM}\right)^{\frac{1}{3}} \tag{S6}$$

B. Thermal activation

Having derived the area per pin for a given applied field, we now follow Ref. 15 to estimate the required activation energy. Consider a domain wall moving along y interacting with a pin,

with interaction energy $V(y)$. The energy is then a sum of the interaction energy and the magnetization energy

$$E = V(y) - 2HMly \tag{S7}$$

where l is the domain wall length and y is the wall displacement. It is not *a priori* known what the form of the interaction potential is, however we can consider some general polynomial expansion $V(y) = a_0 + a_1 y + a_3 y^3 + ...$ where the function is chosen to be odd such that $\mathrm{d}V(y)/\mathrm{d}y$ is symmetrical about $y = 0$ (chosen to be the location of the defect). We follow [15] and choose a potential of the form

$$V(y) = F\left(a + y - \frac{y^3}{3b^2}\right). \tag{S8}$$

Differentiating with respect to y we find that the energy is extremal for

$$y = \pm b\sqrt{1 - \frac{2HMl}{F}} \tag{S9}$$

with an activation energy of

$$E_{\mathrm{act}} = E_{\mathrm{max}} - E_{\mathrm{min}} = \frac{4}{3}\frac{b}{\sqrt{F}}\left(F - 2HlM\right)^{\frac{3}{2}}. \tag{S10}$$

Rearranging, we have

$$2HlM = F\left[1 - \left(\frac{3E_{\mathrm{act}}}{4bF}\right)^{\frac{2}{3}}\right]. \tag{S11}$$

Finally, we rearrange for the coercive field. The length of the domain wall per pin is given by $2L_1$. We thus have

$$l = 2\left(\frac{\gamma}{4\rho HM}\right)^{\frac{1}{3}}. \tag{S12}$$

Subbing into Eqn. S11 and rearranging, we obtain

$$H = \frac{\sqrt{\rho}}{4\sqrt{\gamma}M}F^{\frac{3}{2}}\left[1 - \left(\frac{3E_{\mathrm{act}}}{4bF}\right)^{\frac{2}{3}}\right]^{\frac{3}{2}}. \tag{S13}$$

Assuming thermal activation, i.e. $E_{\mathrm{act}} = k_{\mathrm{B}}T$ and putting the other terms into an effective pinning energy $E_{\mathrm{P}} = k_{\mathrm{B}}T_{\mathrm{P}} = \frac{4bF}{3}$ and the zero temperature coercive field $H_0 = \frac{\sqrt{\rho}}{4M\sqrt{\gamma}}F^{\frac{3}{2}}$ allows us to rewrite the equation as it is used in the main text, i.e.

$$H = H_0\left[1 - \left(\frac{T}{T_{\mathrm{P}}}\right)^{\frac{2}{3}}\right]^{\frac{3}{2}}. \tag{S14}$$

S10. MAGNETOSTRICTION

To detect the small structural changes due to the relative magnetization of the surface layer and bulk, we have used two different approaches which will be described in the following.

A. Magnetostriction loops

We have measured field ramps while recording $z(B_z)$ to search for signatures of the change in magnetization. To this end, we have ramped the field in loops up to a loop field B_L while recording $z(t)$ (fig. S14(a)), which, because the field is ramped in a linear ramp, can be converted to $z(B_z)$ fig. S14(b). To verify that we have reached the fully polarized state at the fields $\pm B_L$, we have recorded tunneling spectra at the fields marked by a star in (a,b), showing here that they are all identical, fig. S14(c). For reference, fig. S14(d) shows a magnetostriction curve recorded for a gold single crystal (Au(111)). The amplitude of the magnetostriction is comparable, although the features deviate in the details. In general, the STM-based magnetostriction curves will contain contributions from heating due to the eddy currents generated while ramping the field, the magnetostriction of the STM head, piezos and tip itself, and the contribution from the sample. It can here be seen that the magnetostriction of the sample, fig. S14(b), is comparable to these contributions. Therefore, in order to be able to detect the structural change associated with the switching of the magnetization of the surface layer requires a more targeted approach. Notably, the magnetization switching of the surface layer is a sudden structural change, rather than a smoothly varying trace as seen in fig. S14(b, d).

B. Detection of magnetostriction jumps in magnetic field ramps

In order to detect the switching of the surface layer, we have ramped the field from a polarized state to a state where only the bulk is switched (fig. S14(e)) before recording a $z(t)$ trace. The state of the surface has been verified from a tunneling spectrum (purple star indicates time when tunneling spectrum is taken). We have then recorded the $z(t)$ (or, equivalently, $z(B_z)$) trace while ramping the field, figs. S14(f,g). For both ramps, there is a small jump, and tunneling

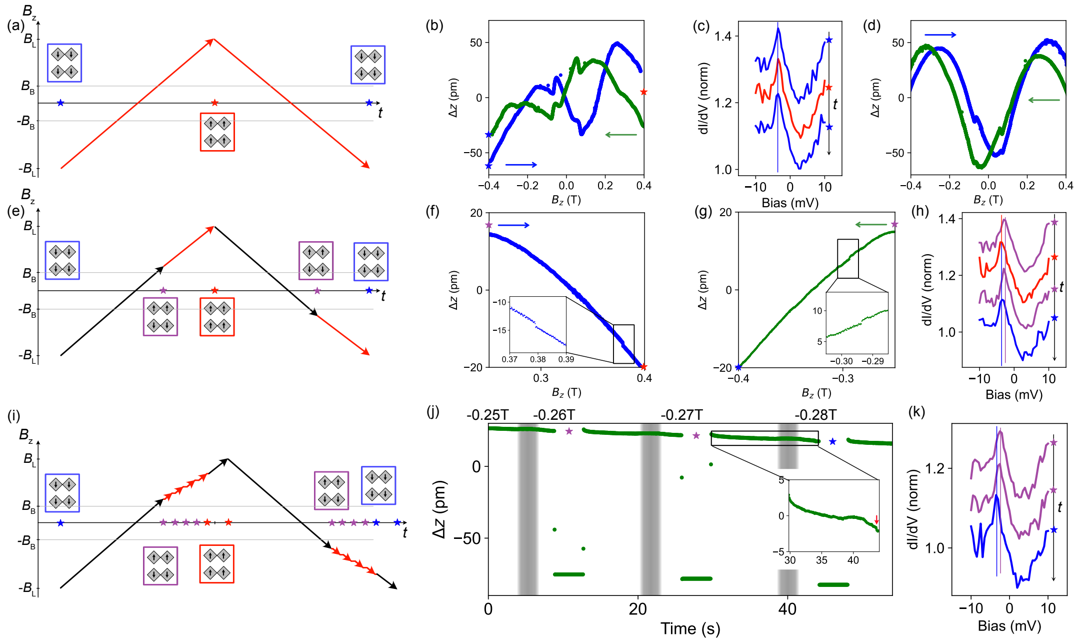

FIG. S14. **Magnetostriction measurements.** (a-d) Magnetostriction loops. (a) Measurement protocol for a full magnetostriction loop. Stars indicate the time when a tunneling spectrum was taken. (b) Full $\Delta z(H)$ trace of $Sr_4Ru_3O_{10}$ while ramping the magnetic field B_z between -0.4T and $+0.4$T and back as sketched in (a). (c) Tunneling spectra recorded before the ramp at $B_z = -B_L = -0.4$T, at the turning point $B_z = B_L = 0.4$T, and after the ramp has finished at $B_z = -B_L = -0.4$T. All three show the peak at the same position, confirming the fully polarized state. (d) $\Delta z(H)$ trace as in (b) for a gold single crystal for reference. (e-h) Magnetostriction loops, where the ramp when the surface switches is recorded separately. (e) Measurement scheme. (f, g) red part of the ramp sketched in (e) where the surface is expected to switch. Insets show jump in $\Delta z(B_z)$ trace when the surface magnetization switches. From spectra (h), we verify that the surface is aligned antiparallel with the bulk before the red section of the ramp in (e) is recorded (purple spectra). (i-k) Measurement to assign jump to surface switching. (i) Measurement scheme to enable assignment of jump. $z(t)$ is continuously recorded while taking spectra and ramping the field alternatingly until the change in the spectrum associated with the surface switching is detected. (j) $z(t)$ trace, the three jumps in $z(t)$ by about -70pm occur whenever a tunneling spectrum is recorded, because it is recorded with a larger setpoint current than the $z(t)$ trace. Between the spectra, the field is ramped up by 10mT before another spectrum is recorded, indicated by grey shaded areas. Here, after three such ramps, a jump in the $z(t)$ trace is detected (see inset, jump marked by red arrow) and (k) the peak in the spectrum has shifted. ($V = 10$mV, $I = 50$pA for b, f, g, j; $V = 10$mV, $I = 450$pA, $V_L = 0.5$mV for spectra in c, h, k)

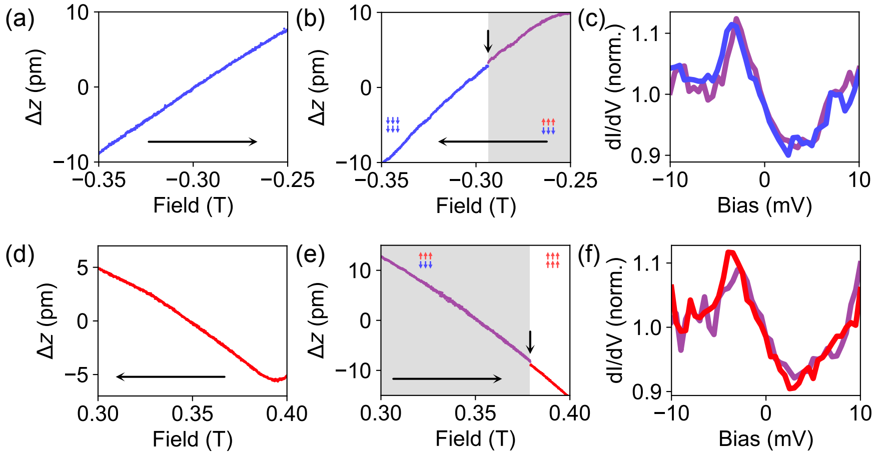

FIG. S15. **Absence of jumps due to the experimental setup.** $z(H)$ traces which (a, d) were acquired when the surface and bulk are already aligned parallel to each other and the applied field, not exhibiting any jumps due to magnetostriction and (b, e) when the surface changes its magnetization, as verified spectroscopically (c, f). The curves in (a, d) are shown in the exact same field range as (b, e), but show no jumps whatsoever ($V_{\mathrm{set}} = 20$mV, $I_{\mathrm{set}} = 50$pA for a, b, d and e; for c, f, the tip has been approached by 43pm from that setpoint to record the spectra, resulting in $I \sim 500$pA at $V \sim 10$mV).

spectra recorded before and after the ramp show small but distinct changes (fig. S14(h)). In principle, also the jump when the bulk switches can be detected (which is then a jump to larger z values), however because this occurs close to the coercive field of the bulk, there are many 'jumps' from domain walls in the bulk moving, and it is therefore more difficult to determine which is related to the sub-surface layer switching.

To demonstrate that in the relevant field range, there are no jumps occurring due to other components of our instrument, we show in fig. S15 for comparison $z(H)$ traces when the surface layer changes its magnetization, as demonstrated by tunneling spectra, and $z(H)$ traces where bulk and surface remain in the same configuration, but in the exact same field range. No jumps can be seen in the ramps where surface and bulk remain in the same configuration.

C. Detection of magnetostriction jumps in time traces

As an alternative way to detect when the surface layer flips its magnetization and narrow down the field range in which this happens, we have continuously recorded a $z(t)$ trace and alternatingly ramped the field by 10mT and taken tunneling spectra to verify the relative orientation of sample and bulk magnetizations (fig. S14(i)). This has been done until the change in magnetization has been detected, enabling to pin down the specific section of the time trace and 10mT ramps at which the switch of magnetization was detected. Fig. S14(j) shows such a trace with the spectra presented in fig. S14(k), where after three ramps of 10mT and taking spectra the surface was found to be switched (parts of the data in fig. S14j, k are shown in fig. 4d of the main manuscript).

The time traces in figs. S14(j) can also be used to extract the apparent barrier height, a measure for the work function of the sample, before and after the surface layer switches. From analyzing the traces, shown in fig. S16 (a, b), we estimate that the work function of the sample is ~ 4.61eV before and after switching. Any change in the work function is at most ~ 5meV, far too small to account of the jump in height observed.

This is also fully consistent with the work function estimated from DFT calculations. Fig. S16(c, d) shows the potential obtained from three layers of Sr_2RuO_4 and $Sr_4Ru_3O_{10}$, respectively, where the difference in the potential is below 5meV and goes in the opposite direction of what would be required to explain the jump in z, i.e. the work function decreases from antiferromagnetic to ferromagnetic configuration, which would result in the tip withdrawing from rather than approaching to the surface.

We note that because the depinning of the domain wall is a statistical process, it does not always happen at the same field, and sometimes occurs with a time delay from when the field was ramped. Occasionally, we have also observed jumps in $z(B_L)$ traces when the surface magnetization had not switched, which is however to be expected when a domain wall passes underneath the tip position deeper inside the material.

While it is inherently easier to detect the jump associated with the magnetization of the surface layer aligning with the field, i.e. switching from being antiparallel to the bulk to being parallel, it is possible to also observe the jump when the bulk switches and hence the configu-

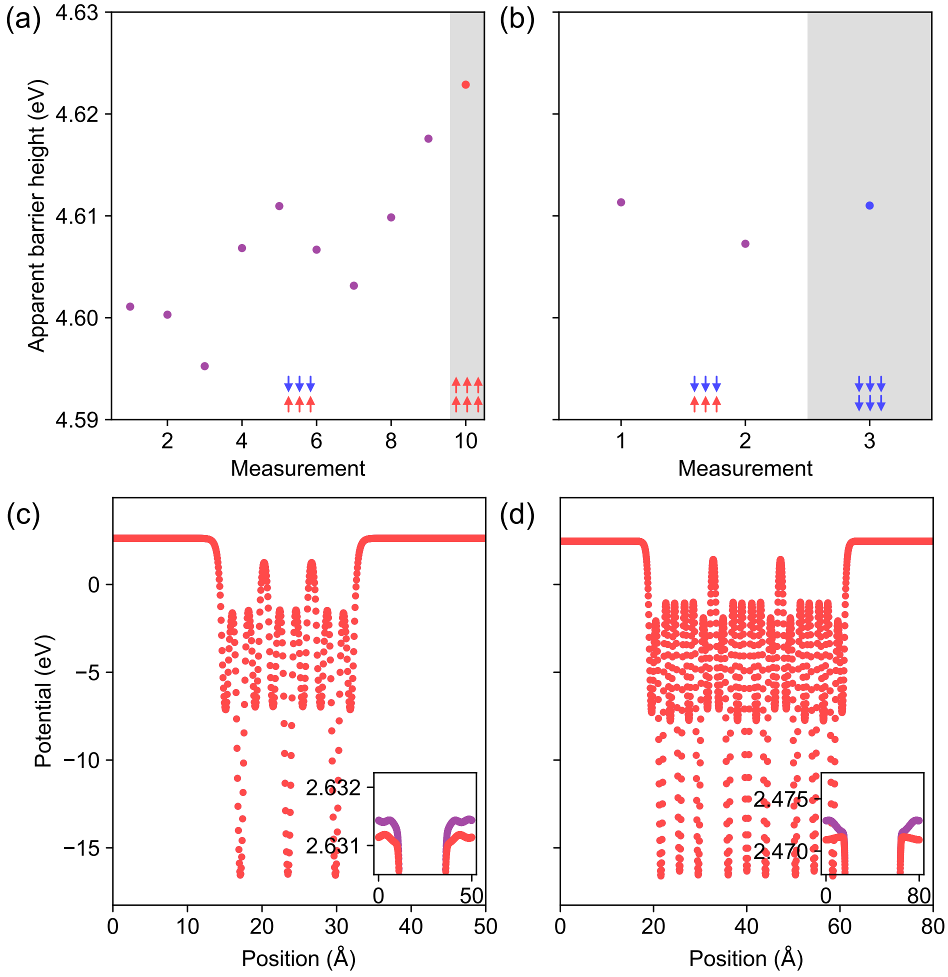

FIG. S16. **Workfunction for ferromagnetic and antiferromagnetic configuration.** (a, b) Apparent barrier height extracted before spectrum is taken to verify magnetization direction obtained from $z(t)$ traces as shown in fig. S14(j). The color of the points and grey shading highlight the magnetic configuration, purple points on white background represent antiferromagnetic configuration, red/blue points on grey shaded background ferromagnetic configuration. (c, d) DFT calculation of the Hartree potential, from which the change in work function between the ferromagnetic and antiferromagnetic configurations can be extracted. The change in potential is below 5meV and in the opposite direction to contribute to the jump in the tip height z when the configuration changes.

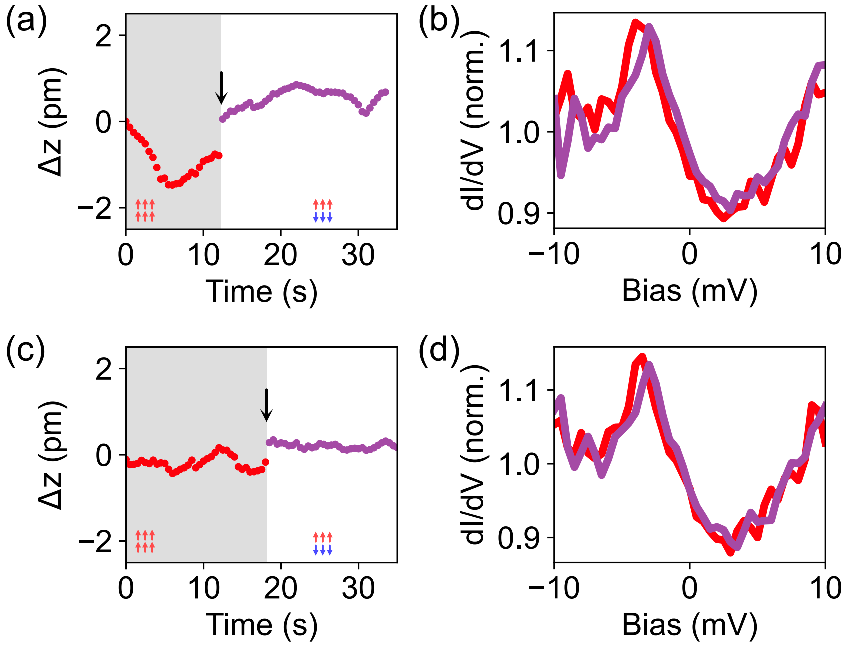

FIG. S17. **Jumps detected when the sub-surface layer switches magnetization.** (a, c) $z(t)$ traces recorded while the magnetic field is ramped by 10mT from -0.16T to -0.17T; and, in a separate loop, (c) from -0.15T to -0.16T (V_{set} = 10mV, I_{set} = 50pA). (b, d) show the corresponding spectra recorded directly before and after the traces in (a) and (c), respectively (V_{set} = 10mV, I_{set} = 450pA, V_{L} = 0.5mV). Note that here the jump in (a) and (c) is in the opposite direction compared to fig. 4 in the main manuscript and the spectra in (b) and (d) also change the opposite way, i.e. the peaks move closer to the Fermi energy across the jump, because here, the configuration changes from ferromagnetic to antiferromagnetic.

ration of surface and bulk changes from parallel to antiparallel, with a slight *expansion* of the surface layer. Fig. S17 shows two such curves, where we ramped the magnetic field by 10mT and could clearly observe a jump in the $z(t)$ trace recorded while ramping when the *bulk* changes its magnetisation as evidenced by tunneling spectra.

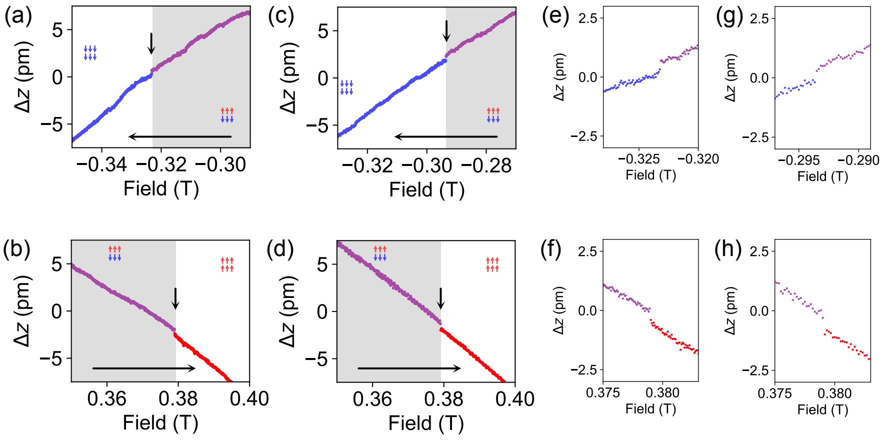

FIG. S18. **Jumps detected with different bias voltages.** (a-h) $z(H)$ traces recorded while the magnetic field is ramped, switching the surface magnetization. Panels (a) and (b) have been recorded with a bias voltage $V_{\mathrm{set}} = 10\mathrm{mV}$ and (c, d) with $V_{\mathrm{set}} = 20\mathrm{mV}$. The jumps are of almost identical height. Panels (e-h) show close-ups of panels (a-d) around the field range of the jumps. All traces recorded with $I_{\mathrm{set}} = 50\mathrm{pA}$.

D. Absence of bias-dependence in jumps

In principle, a change in the density of states could also result in a change in apparent height that would be detected as magnetostriction in STM, because the tip sample distance depends on the density of states. We expect this effect to be minimal because the change in tunneling spectra, and, indeed, the electronic structure between the two magnetic configurations is minimal. We show here magnetostriction traces that support this conclusion from experiment. Fig. S18 shows magnetostriction curves $z(H)$ acquired at bias voltages $V_{\mathrm{set}} = 10\mathrm{mV}$ and $V_{\mathrm{set}} = 20\mathrm{mV}$, showing that the jumps have the same height. This is also supported from tunneling spectra acquired in a wider bias range, see Fig. S19, which show practically no difference in differential conductance at positive bias voltages, and at negative bias voltages the main difference is indeed the change in peaks $\mathrm{P_I}$.

To determine whether there are changes in the differential conductance in a wider energy range that could lead to changes in the apparent height and hence be detected as a contribution to the magnetostriction, we measured tunneling spectra in the range ±40 mV for both parallel

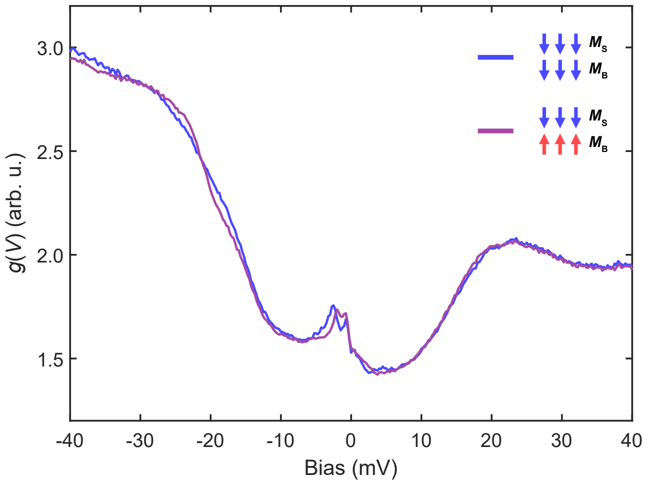

FIG. S19. **Differential conductance spectroscopy in ±40 mV energy range for parallel and antiparallel surface and bulk magnetizations.** The blue curve shows the tunneling spectra at −0.1 T after polarizing the overall sample magnetization with −10 T, so that both surface and bulk magnetizations ate pointing down. The purple curve shows the tunneling spectrum at 0.15 T, when the bulk magnetization has flipped in relation to the surface. While the energy shift of peak P_I is visible, there is almost no change in differential conductance at bias voltages above the Fermi energy (0V). Both curves are the result of averaging of differential conductance spectra recorded on an 16×16 grid over a (1.5×1.5) nm^2 area ($V_{set} = 40$ mV, $I_{set} = 500$ pA, $V_L = 250$ μV).

and antiparallel configurations of surface and bulk magnetizations. Figure S19 shows tunneling spectra taken at −0.10 T after polarizing the overall magnetization of the sample in −10 T, blue curve, showing peak P_I at it's characteristic position for parallel surface and bulk magnetizations. The purple curve in Figure S19 shows the tunneling spectra after ramping to 0.15 T, where the bulk magnetization is flipped in relation to the surface. Both measurements were taken with the same tip in the same area of the sample. There is practically no change in the differential conductance at positive bias voltages, and changes to the differential conductance are limited to a bias range of $-5 \ldots 0$mV.

[1] R. Cardias, A. Szilva, A. Bergman, I. Di Marco, M. I. Katsnelson, A. I. Lichtenstein, L. Nordström, A. B. Klautau, O. Eriksson, and Y. O. Kvashnin. The Bethe-Slater curve revisited; new insights from electronic structure theory. *Sci Rep*, 7(1):4058, June 2017. ISSN 2045-2322. doi:10.1038/s41598-017-04427-9. URL https://www.nature.com/articles/s41598-017-04427-9.

[2] Carolina A. Marques, Philip A. E. Murgatroyd, Rosalba Fittipaldi, Weronika Osmolska, Brendan Edwards, Izidor Benedičič, Gesa-R. Siemann, Luke C. Rhodes, Sebastian Buchberger, Masahiro Naritsuka, Edgar Abarca-Morales, Daniel Halliday, Craig Polley, Mats Leandersson, Masafumi Horio, Johan Chang, Raja Arumugam, Mariateresa Lettieri, Veronica Granata, Antonio Vecchione, Phil D. C. King, and Peter Wahl. Spin-orbit coupling induced Van Hove singularity in proximity to a Lifshitz transition in $Sr_4Ru_3O_{10}$. *npj Quantum Mater.*, 9(1):35, April 2024. ISSN 2397-4648. doi:10.1038/s41535-024-00645-3. URL https://www.nature.com/articles/s41535-024-00645-3.

[3] G. Gebreyesus, Prosper Ngabonziza, Jonah Nagura, Nicola Seriani, Omololu Akin-Ojo, and Richard M. Martin. Electronic structure and magnetism of the triple-layered ruthenate $Sr_4Ru_3O_{10}$. *Phys. Rev. B*, 105(16):165119, April 2022. ISSN 2469-9950, 2469-9969. doi:10.1103/PhysRevB.105.165119. URL https://link.aps.org/doi/10.1103/PhysRevB.105.165119.

[4] Arash A. Mostofi, Jonathan R. Yates, Giovanni Pizzi, Young-Su Lee, Ivo Souza, David Vanderbilt, and Nicola Marzari. An updated version of wannier90: A tool for obtaining maximally-localised Wannier functions. *Computer Physics Communications*, 185(8):2309–2310, August 2014. ISSN 00104655. doi:10.1016/j.cpc.2014.05.003. URL https://linkinghub.elsevier.com/retrieve/pii/S001046551400157X.

[5] Anirudh Chandrasekaran, Luke C. Rhodes, Edgar Abarca Morales, Carolina A. Marques, Phil D. C. King, Peter Wahl, and Joseph J. Betouras. Engineering higher order Van Hove singularities in two dimensions: the example of the surface layer of Sr_2RuO_4, October 2023. URL http://arxiv.org/abs/2310.15331. arXiv:2310.15331 [cond-mat].

[6] Veronica Granata, Lucia Capogna, Manfred Reehuis, Rosalba Fittipaldi, Bachir Ouladdiaf, Sandro Pace, Mario Cuoco, and Antonio Vecchione. Neutron diffraction study of triple-layered $Sr_4Ru_3O_{10}$. *J. Phys.: Condens. Matter*, 25(5):056004, February 2013. ISSN 0953-8984, 1361-648X. doi:10.1088/0953-8984/25/5/056004. URL https://iopscience.iop.org/article/10.1088/0953-8984/25/5/056004.

[7] H. Zheng, W. H. Song, J. Terzic, H. D. Zhao, Y. Zhang, Y. F. Ni, L. E. DeLong, P. Schlottmann, and

G. Cao. Observation of a pressure-induced transition from interlayer ferromagnetism to intralayer antiferromagnetism in $Sr_4Ru_3O_{10}$. *Phys. Rev. B*, 98(6):064418, August 2018. ISSN 2469-9950, 2469-9969. doi:10.1103/PhysRevB.98.064418. URL https://link.aps.org/doi/10.1103/PhysRevB.98.064418.

[8] L. Capogna, V. Granata, B. Ouladdiaf, J.A. Rodriguez-Velamazan, R. Fittipaldi, and A. Vecchione. Layer dependent antiferromagnetism in the $Sr_4Ru_3O_{10}$ ruthenate at the metamagnetic-like transition. *Journal of Magnetism and Magnetic Materials*, 493:165698, January 2020. ISSN 03048853. doi:10.1016/j.jmmm.2019.165698. URL https://linkinghub.elsevier.com/retrieve/pii/S0304885319311035.

[9] Izidor Benedičič, Masahiro Naritsuka, Luke C Rhodes, Christopher Trainer, Yoshiko Nanao, Aaron B Naden, Rosalba Fittipaldi, Veronica Granata, Mariateresa Lettieri, Antonio Vecchione, and Peter Wahl. Interplay of ferromagnetism and spin-orbit coupling in $Sr_4Ru_3O_{10}$. *Physical Review B*, 106:L241107, 2022. doi:10.1103/PhysRevB.106.L241107. URL https://journals.aps.org/prb/abstract/10.1103/PhysRevB.106.L241107.

[10] Carolina A Marques, Luke C Rhodes, Rosalba Fittipaldi, Veronica Granata, Chi Ming Yim, Renato Buzio, Andrea Gerbi, Antonio Vecchione, Andreas W Rost, and Peter Wahl. Magnetic-Field Tunable Intertwined Checkerboard Charge Order and Nematicity in the Surface Layer of Sr_2RuO_4. *Advanced Materials*, 33:2100593, 2021. ISSN 10.1002/adma.202100593. doi:10.1002/adma.202100593. URL https://onlinelibrary.wiley.com/doi/10.1002/adma.202100593.

[11] Carolina A. Marques, Luke C. Rhodes, Izidor Benedičič, Masahiro Naritsuka, Aaron B. Naden, Zhiwei Li, Alexander C. Komarek, Andrew P. Mackenzie, and Peter Wahl. Atomic-scale imaging of emergent order at a magnetic field–induced Lifshitz transition. *Sci. Adv.*, 8(39):eabo7757, September 2022. ISSN 2375-2548. doi:10.1126/sciadv.abo7757. URL https://www.science.org/doi/10.1126/sciadv.abo7757.

[12] P. Gaunt. Ferromagnetic domain wall pinning by a random array of inhomogeneities. *Philosophical Magazine B*, 48(3):261–276, September 1983. ISSN 1364-2812, 1463-6417. doi:10.1080/13642818308228288. URL https://www.tandfonline.com/doi/full/10.1080/13642818308228288.

[13] W. Yan, L.C. Phillips, and N.D. Mathur. Temperature-Dependent Mechanism of Magnetization Reversal in the Spintronic Electrode Material $La_{0.67}Sr_{0.33}MnO_3$. *Phys. Rev. Applied*, 18(5):054084, November 2022. ISSN 2331-7019. doi:10.1103/PhysRevApplied.18.054084. URL https://link.aps.org/doi/10.1103/PhysRevApplied.18.054084.

[14] P. Gaunt. The frequency constant for thermal activitation of a ferromagnetic domain wall. *Journal of*

Applied Physics, 48(8):3470–3474, August 1977. ISSN 0021-8979, 1089-7550. doi:10.1063/1.324195. URL http://aip.scitation.org/doi/10.1063/1.324195.

[15] P. Gaunt and G. J. Roy. Magnetic viscosity in ferromagnets: II. Co_5Ce permanent magnet particles. *Philosophical Magazine*, 34(5):781–788, November 1976. ISSN 0031-8086. doi:10.1080/14786437608222050. URL http://www.tandfonline.com/doi/abs/10.1080/14786437608222050.
